# A Polymer-Based Metallurgical Route to Produce Aluminum Metal-Matrix Composite with High Strength and Ductility

**DOI:** 10.3390/ma17010084

**Published:** 2023-12-23

**Authors:** Bindu Gutta, Prashant Huilgol, Chandra S. Perugu, Govind Kumar, S. Tejanath Reddy, Laszlo S. Toth, Olivier Bouaziz, Satish V. Kailas

**Affiliations:** 1 Centre for Product Design and Manufacturing, Indian Institute of Science, Bangalore 560012, India; guttabindu@iisc.ac.in (B.G.); govindkumar@iisc.ac.in (G.K.); satvk@iisc.ac.in (S.V.K.); 2 Department of Mechanical Engineering, Indian Institute of Science, Bangalore 560012, India; hprashant@iisc.ac.in; 3 Department of Materials Engineering, Indian Institute of Science, Bangalore 560012, India; pcrnish@gmail.com (C.S.P.); tejanaths@iisc.ac.in (S.T.R.); 4 Laboratory of Excellence on Design of Alloy Metals for Low-Mass Structure (Labex-DAMAS), Lorraine University, 57070 Metz, France; 5 Laboratoire d’Etude des Microstructures et de Mécanique des Matériaux, UMR 7239, CNRS/Université de Lorraine, 57070 Metz, France; olivier.bouaziz@univ-lorraine.fr; 6 Institute of Physical Metallurgy, Metal-Forming and Nanotechnology, University of Miskolc, 3515 Miskolc, Hungary

**Keywords:** metal–matrix composite, aluminum, polymer-derived ceramic, grain stability, high strength, ductility

## Abstract

In this investigation, an attempt was made to develop a new high-strength and high-ductility aluminum metal–matrix composite. It was achieved by incorporating ceramic reinforcement into the metal which was formed in situ from a polymer by pyrolysis. A crosslinked PMHS polymer was introduced into commercially pure aluminum via friction stir processing (FSP). The distributed micro- and nano-sized polymer was then converted into ceramic particles by heating at 500 °C for 10 h and processed again via FSP. The produced composite showed a 2.5-fold increase in yield strength (to 119 MPa from 48 MPa) and 3.5-fold increase in tensile strength (to 286 MPa from 82 MPa) with respect to the base metal. The ductility was marginally reduced from 40% to 30%. The increase in strength is attributed to the grain refinement and the larger ceramic particles. High-temperature grain stability was obtained, with minimal loss to mechanical properties, up to 500 °C due to the Zenner pinning effect of the nano-sized ceramic particles at the grain boundaries. Fractures took place throughout the matrix up to 300 °C. Above 300 °C, the interfacial bonding between the particle and matrix became weak, and fractures took place at the particle–matrix interface.

## 1. Introduction

Aluminum alloys are widely used in the aerospace and automotive industries due to their high specific strength, corrosion resistance, good electrical and thermal conductivity, weldability, creep, and damage tolerance. Alloys are designed for specific applications by adding required alloying elements and using suitable processing conditions. These are industrially classified based on the major alloying element as 1XXX (pure Al min 99%), 2XXX (Al-Cu), 3XXX (Al-Mn), 4XXX (Al-Si), 5XXX (Al-Mg), 6XXX (Al-Mg-Si 1%), 7XXX (Al-Zn-Mg-Cu), and 8XXX (Al-other element) series. Within each of these classes, the properties vary widely depending on the alloying chemistry and processing conditions. This means that there are more than 100 types of Al alloys being used. On one hand, the use of aluminum alloys reduces energy consumption due to their light weight, while the extraction of aluminum from its ore is highly energy-intensive. Aluminum extraction from ore is one of the largest sources of greenhouse gas (GHG) emissions [1,2]. The annual global production of aluminum is close to 6 × 10^7^ tons/year [2]. The energy spent in the extraction of aluminum alloy is 190–230 MJ/kg, and the associated CO_2_ footprint is 11–13 kg/kg. Meanwhile, for the recycling of aluminum, the embodied energy is 22–39 MJ/kg, and the CO_2_ footprint is only 1.9–2.3 kg/kg [2]. This implies that the primary production of the metal generates about 12–15 tons of GHG emissions per ton of metal produced [2,3,4,5]. A major part of these GHG emissions comes from using energy from fossil fuels [5]. Globally, aluminum production consumes 1% of total energy and produces 3% of total GHG emissions [6]. Recycling reduces energy consumption by more than 90% compared to energy used to make aluminum from bauxite [1]. It involves remelting from scrap. Recycling expends 2.8 kWh/kg due to aluminum’s low melting point, while primary synthesis expends 45 kWh/kg due to the large enthalpy of oxide [7]. Therefore, recycling can shift the energy balance towards higher sustainability. However, recycling scraps from many of these alloy systems poses a major challenge. The problem today is to develop a new alloy design strategy that is compatible with recycling.

One approach is to use ceramic–metal matrix composites. The reinforcement can be separated out by melting the composite. Traditionally, ceramic reinforced composites are produced via [8,9,10]:

- the powder metallurgy route (mechanical alloying and densification),
- internal oxidation,
- the molten metal route via stir-casting methods or impregnation.

The disadvantages of the casting route are agglomeration, poor wettability, and poor interfacial bond strength. The energy input to break the particles down to nanoscale is very high in the powder metallurgy route and is cost-ineffective. The ceramic reinforcements are expected to provide high strength and high elastic modulus while the aluminum matrix provides high ductility and toughness. While the micro-sized reinforcements provide enhanced strength by acting as barrier to dislocation movement, a significant drop in ductility is expected. Alternatively, the nano-sized reinforcements provide both higher strength and ductility, depending on their size and distribution, provided a good bonding between the particle and the matrix is ensured.

We intend to use a polymer-derived ceramic (PDC) as a reinforcement. This is a class of polymers that convert to ceramic when heated to high temperatures [11,12,13,14,15,16]. To make the Al-PDC composite, the friction stir-processing (FSP) route seems to be a promising technique. FSP has emerged as an effective process in producing surface and bulk metal matrix composites [17]. During FSP, the material undergoes severe plastic deformation resulting in grain refinement of the matrix along with breaking and mixing of particles. Superior mechanical properties are obtained when the particles are stable, fine, and evenly distributed along with good interfacial bonding between the particles and the matrix. One of the problems associated with the mixing of hard ceramic particles via FSP is tool wear and life. Moreover, the energy input to reduce the particle size from micro- to nanoscale increases enormously with the reduction in particle size. This is expected, since the defect density decreases in a particle as it becomes smaller. Some of these limitations can be overcome by using a PDC [18,19,20,21]. The polymer is soft and can be easily broken into finer particles without any tool wear. Further, the polymer can be converted into ceramic via the process of pyrolysis. Pyrolysis involves heating the polymer to a certain temperature over time to convert it to a ceramic. Recent studies on copper polymer-derived ceramic composite have shown a five-fold increase in hardness compared to commercially pure copper. Moreover, the ceramic particles were significantly reduced to the size of 10–30 nm with zero tool wear [18,19]. One important feature of severe plastic deformation (SPD) techniques, including FSP, is the significant amount of grain refinement and increased defect density. While it is very beneficial for increasing material strength, it also leads to a high amount of stored energy in the material. This affects microstructural instability and an increased tendency for grain growth at high temperatures. Generally, ultrafine-grained materials undergo abnormal grain growth at higher temperatures. The distribution of nano-sized particles within the grains and along the grain boundaries can influence both mechanical properties and thermal stability at higher temperatures.

In this regard, an attempt is made in the present investigation to show that an Al-PDC composite can be produced with improved mechanical properties and thermal stability. Therefore, by controlling the size, distribution, and volume fraction of PDC reinforcements in the aluminum matrix, it is possible to produce a single class of composite with the desired Young’s modulus, strength, ductility and thermal stability. Such a composite has the potential to replace some of the existing classes of aluminum alloys, thereby increasing recyclability and sustainability.

## 2. Materials and Methods

Commercially available pure aluminum plate (99.6% purity) with dimensions of 210 mm × 75 mm × 6 mm was used as the matrix material for the study. A silicon-based polymer poly-methyl-hydro siloxane (PMHS) was used as the precursor for the polymer reinforcement. The procured polymer precursor was in liquid form with an average molecular weight in the range of 1700 to 3200 g mol^−1^. The precursor chain comprises Si, O, C, and H with the chemical formula (CH_3_)3SiO [(CH_3_)HSiO]nSi(CH_3_). The liquid precursor was crosslinked with 4-diazabicyclo [2.2.2] octane (DABCO) to obtain a rigid polymer [22]. The rigid polymer was further ground into a fine powder. Grooves 3 mm wide and 4 mm deep were machined on the aluminum plates and filled with polymer powder. Next, the groove filled with powder was sealed with a 2 mm-thick cover strip made of the same material as the plate.

Friction stir-processing (FSP) was carried out on a five-axis friction stir welding machine. A hot die steel tool with a frustum shape and a pin diameter varying from 10 mm at the top to 8 mm at the bottom and a length of 5 mm, with left-handed threads, was used. The tool shoulder diameter was 30 mm. The processing was carried out with a tool rotation of 1200 rpm and a traverse speed of 25 mm/min. A 2° tool tilt was provided. Seven passes of FSP with a 30 min break between each pass were carried out to disperse the polymer powder into the aluminum matrix. Next, the processed plate was kept in a furnace maintained at 500 °C for 10 h for the pyrolysis of the polymer. During this stage, the polymer was converted into ceramic. The plate was then removed from the furnace and cooled in air. After pyrolysis, the plate was again processed with the same process parameters to disperse the ceramic particles and remove the porosity formed during pyrolysis.

For mechanical and microstructural characterization, samples were machined via EDM. The ASTM E8 standard [23] was followed for tensile testing. The specimens for tensile testing having 6 mm gauge length were sliced along the tool travel direction. Ambient and high-temperature tensile tests were performed on a UTM machine at a strain rate of 10^−3^ s^−1^. Three samples were tested for each condition. High-temperature tensile tests were carried out in the temperature range of 100 °C to 500 °C. Microstructural investigations were carried out using scanning electron microscopy (SEM) equipped with EBSD and transmission electron microscopy (TEM). Samples for SEM were prepared via standard metallographic polishing. Samples for EBSD were prepared via electropolishing with A2 electrolyte (perchloric acid 7.9%, distilled water 7.2%, ethanol 74.6%, butoxyethanol 10.2%). For the base material and the processed base metal, the step size of 4 µm was used. For the composite, a step size of 200 nm was used in EBSD measurements. Samples for TEM analysis were prepared by first cutting a 500 µm-thick slice from the processed zone. The slice was then carefully polished mechanically to a thickness of 80 µm. Next, a 3 mm disc was punched and disc-ground. The samples were further thinned down to electron transparency via ion-polishing. The sizes of the particles were measured using image analysis software ImageJ (version 1.54g) from several SEM and TEM micrographs taken at various magnifications. To study the Bauschinger effect, cyclic tension tests, followed by compression tests, were carried out as per the ASTM E606 standard [24]. The tests were conducted over six cycles at a strain rate of 10^−4^ s^−1^ and constant strain amplitude. A 12.5 mm strain gauge (extensometer) was used for measuring and controlling the strain in the specimen.

## 3. Results and Discussion

### 3.1. The Al-PDC Composite

**Figure 1:**
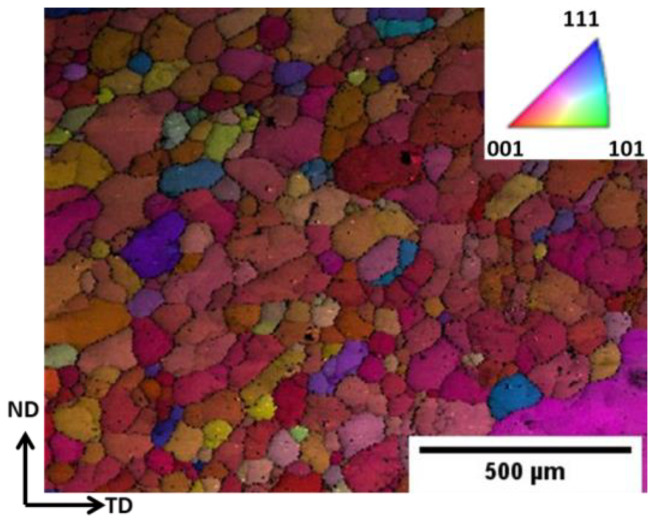
SEM-EBSD micrograph of the base metal having an average grain size of 100 μm.

Figure 1 represents SEM-EBSD data. The micrograph shows the inverse pole figure (IPF) map of the normal direction of the base metal. The base metal was received in a rolled condition. The average grain size was around 100 µm. The composite was prepared by first incorporating the polymer via FSP. The composite was later heated in a furnace for the pyrolysis of the polymer to take place. This process converted the polymer into ceramic. These ceramic particles were amorphous and incoherent with the matrix. These characteristics of polymer-derived ceramics are examined in detail in refs. [15,16]. Crystallization of SiOC ceramics can take place only above 1300 °C, which cannot be reached in aluminum. Further characteristics of the PDC in our material were investigated via SEM and TEM and will be presented later in the article.

**Figure 2:**
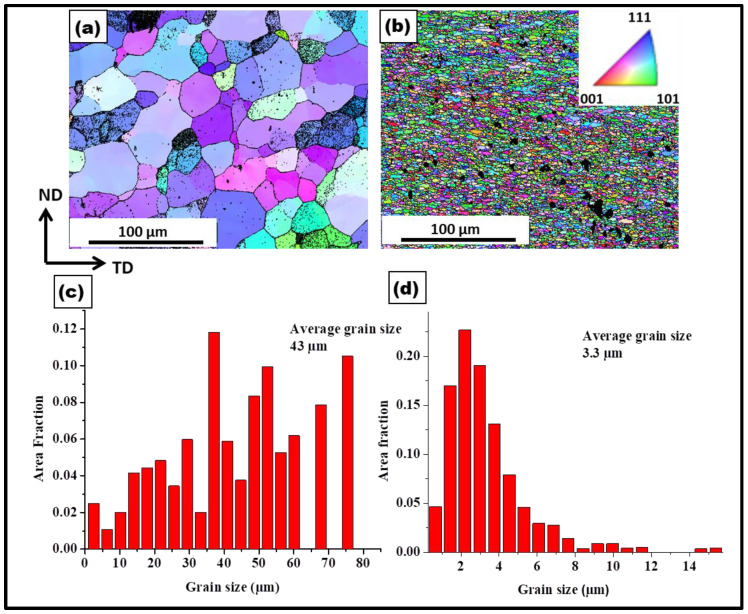
SEM-EBSD micrograph of aluminum (**a**): processed without a PDC, (**b**): composite, (**c**) grain size distribution of aluminum processed without a PDC with an avg. grain size of 43 µm, (**d**) grain size distribution of composite with an avg. grain size of 3.3 µm.

Figure 2 shows SEM-EBSD micrographs of the samples processed with and without PDC reinforcement. The average grain size measured in the processed sample was around 43 µm, and for the composite, it was around 3.3 µm. A significant amount of grain refinement was observed with the addition of the PDC. The ceramic particles size ranged from micron to submicron sizes as observed in the un-indexed regions in Figure 2b.

**Figure 3:**
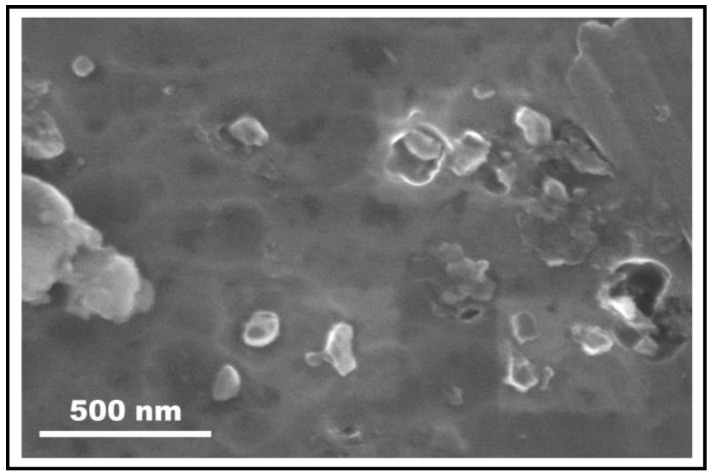
SEM micrograph of FSP-processed aluminum composite showing presence of ceramic particles with sizes less than 200 nm.

**Figure 4:**
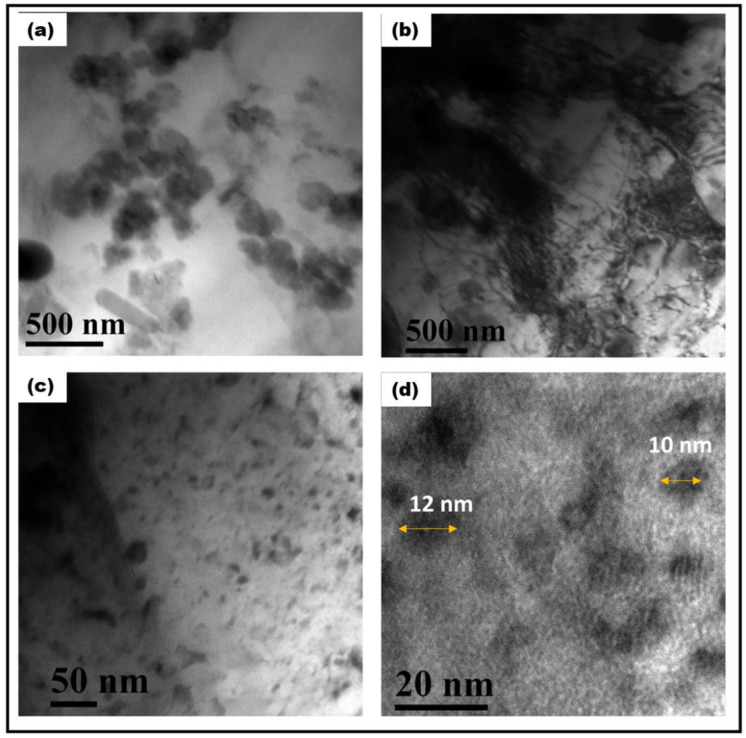
(**a**): Bright-field TEM micrograph of FSP-processed Al composite; particles are seen in dark contrast, while the matrix is bright contrast. (**b**): Bright-field TEM micrograph showing dislocation substructure. (**c**): FSP-processed composite showing fine particles. (**d**): High-magnification micrograph showing particles in the size range of 10–15 nm.

Figure 3 displays FSP-processed Al composite showing PDC reinforcements. The size of the ceramic particles was measured in the range of 100–300 nm. This aspect was further confirmed via TEM studies. Figure 4a shows a bright-field TEM micrograph of the FSP-processed Al composite. Ceramic particles with dark contrast are seen in the Al matrix. The size of the particles measured was in close agreement with the SEM studies.

**Figure 5:**
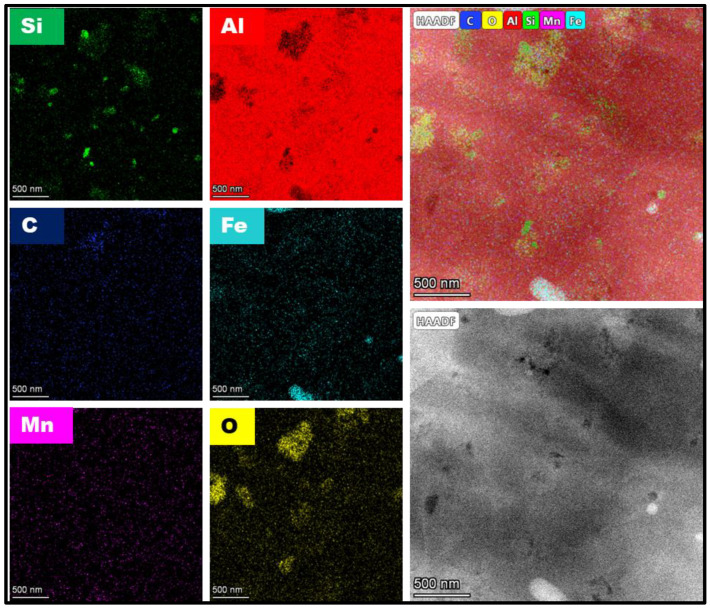
TEM-EDS area mapping of the ceramic reinforcements in the matrix.

Dislocation substructures formed during the process are shown in Figure 4b. Dislocations are seen to be arranged in cell walls. The TEM micrographs shown in Figure 4 reveal that the particle size distribution of the ceramic particles ranged from micron to nano-size, with the smaller particles being in the range of 10–40 nm. Next, TEM-EDS area mapping confirms the dark contrasted particles to be ceramic as presented in Figure 5. Additionally, the absence of diffraction contrast from the ceramic particles indicates that the ceramic was amorphous.

**Figure 6:**
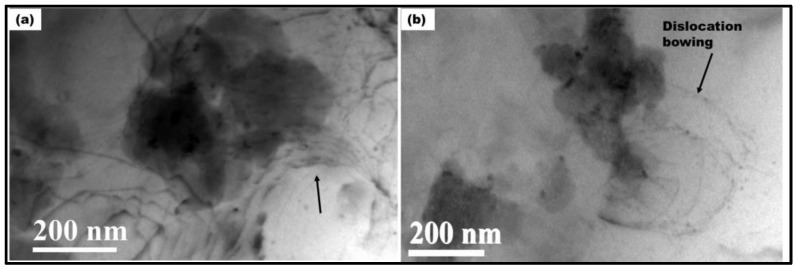
(**a**,**b**) Bright-field TEM micrograph showing the formation of dislocation loops. Dislocation bowing near the particles is marked with the arrow.

During FSP, material flow takes place at high strain rates and temperatures [17]. Finite element simulations show that the amount of plastic strain during FSP can go very high, up to 17 on the von Mises equivalent strain scale [25]. The state of deformation, the extent of the process of recovery, and recrystallization strongly depend on the nature of the second-phase particles in the matrix. The ceramic particles have an influence on recrystallization kinetics and subsequent grain growth. During deformation, the ceramic particles tend to be rigid, while the matrix becomes soft and flows plastically. The strain gradient is accommodated via additional generation of dislocations at the particle–matrix interface. A higher dislocation density is expected in the vicinity of the particles due to GNDs (geometrically necessary dislocations) and dislocation particle interaction. This condition leads to particle-assisted nucleation of recrystallization [26]. The fact that significant grain refinement is observed via the incorporation of the particles suggests that the particles are essentially incoherent with the matrix, unlike weak coherent particles, which deform at the same rate as the matrix, and no GNDs are produced. Additionally, moving dislocations bow and loop around the smaller particles which act as Frank–Reed sources for further generation of dislocations. This is illustrated in Figure 6.

Overall, the rate of dislocation generation was higher in the composite as compared to the base metal. However, under greater strains, annihilation and rearrangement of dislocations took place via the process of dynamic recovery due to the high stacking fault energy of Al. The particles affected the dynamic recovery by restricting the motion of dislocations and influencing the cell/subgrain structure.

**Figure 7:**
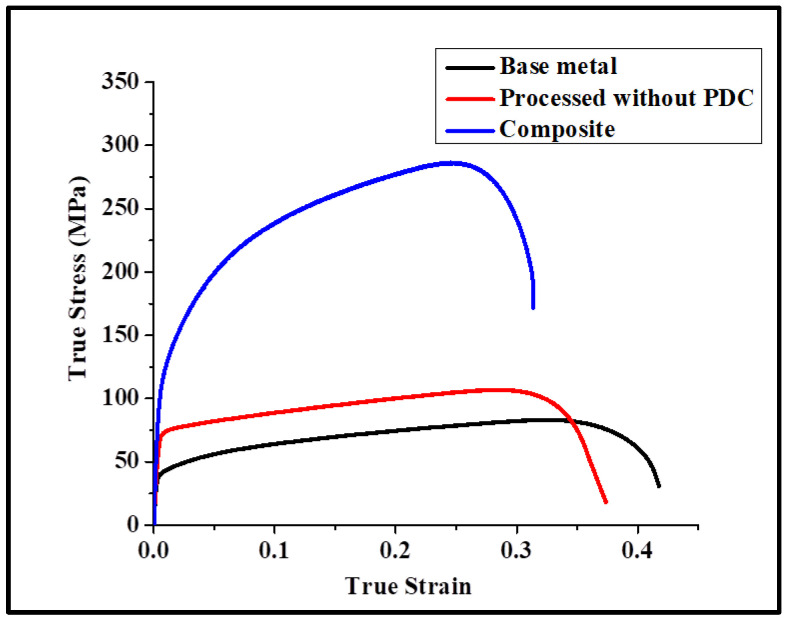
Tensile stress–strain curves at room temperature of the base metal, the processed base metal, and the composite.

Figure 7 shows the tensile stress–strain curves for the parent metal, for the processed metal without PDC, and for the composite, respectively. The composite showed a 250% increase in strength compared to the base metal. A 25% drop in ductility was measured when compared with the processed metal without the PDC. The yield strength of the composite was measured to be around 119 ± 7 MPa, the ultimate tensile strength 286 ± 8 MPa, and the ductility 30 ± 4%. 

**Figure 8:**
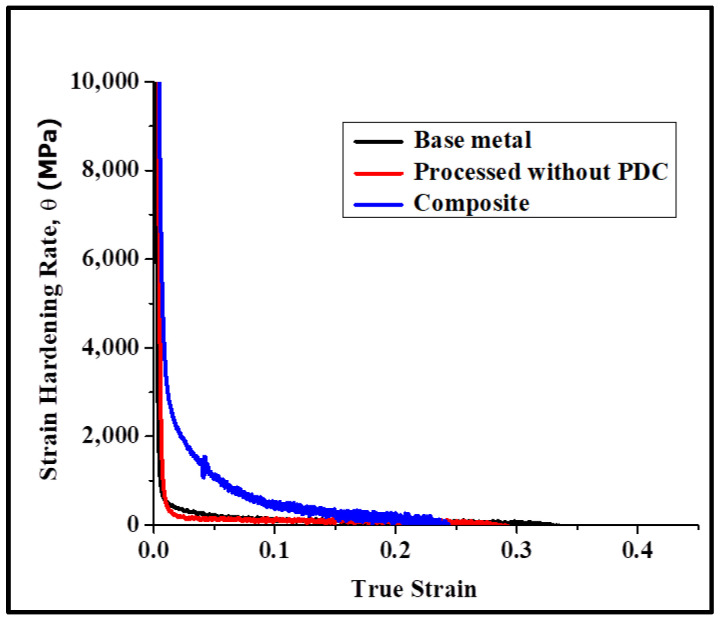
Strain hardening rate vs. true strain for the base metal, processed without PDC and the composite. The composite exhibits significant strain hardening.

**Figure 9:**
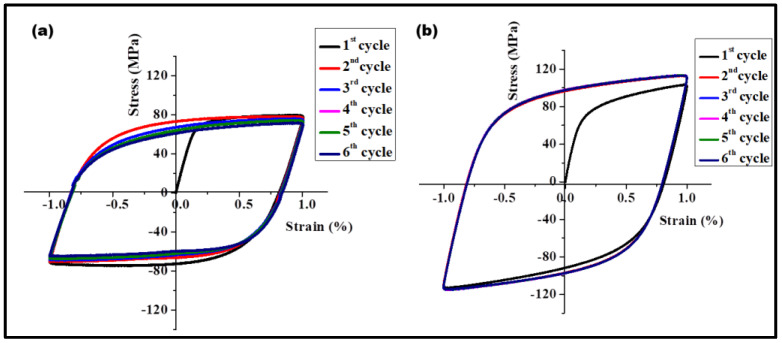
Bauschinger tests (tension followed by compression) for (**a**) base metal (**b**) composite at ±1% strain amplitude and strain rate of 10^−4^ s^−1^.

The true stress–strain curve is further analyzed to understand the strain hardening behavior. The strain hardening rate is plotted as the function of true strain for both the base metal and composite and is shown in Figure 8. The composite exhibits a higher strain hardening rate. The higher strain hardening rate for the composite is associated with dislocation accumulation via generation and multiplication from a Frank–Reed source (due to non-shearable particles) and grain boundaries. Dynamic recovery takes place via the mechanisms of annihilation of dislocations of opposite signs, cross-slip and climb [27,28]. Additionally, the formation of dislocation loops around the non-shearable particles has implications for the local storage of elastic energy. This aspect is confirmed by the presence of the Bauschinger effect. Figure 9 shows the results of Bauschinger tests for ±1% strain amplitude.

The internal stress is known as the back stress defined as $\sigma_{back}=\left(\sigma_{F}-\sigma_{R}\right)\;/2$, where $\sigma_{F}$ is the forward yield stress and $\sigma_{R}$ is the yield stress in the reverse loading. The back stress $\sigma_{back}$ measured for the base material was 15 MPa, and for the composite, it was 52 MPa. The plastic stress and strain distributions are non-uniform, which gave rise to internal stresses. The internal stresses reduced the yield stress during load reversal. There are several mechanisms responsible for back stress [29,30,31,32,33]. Deformation incompatibility among the grains in the polycrystal is compensated via the generation of GNDs, which give rise to internal stresses. Polycrystal simulations also show the Bauschinger effect without accounting for non-shearable particles [34,35], due to the effect of kinematic hardening in crystal plasticity, which is appears naturally in polycrystalline materials. Indeed, the base metal also shows a significant Bauschinger effect (seen in Figure 9a). However, it is clear that the presence of the particles increases it significantly, already causing deviation from linear elasticity before fully reversing the loading direction (see Figure 9b).

### 3.2. High-Temperature Tensile Tests and Thermal Stability

#### 3.2.1. High-Temperature Tensile Test

**Figure 10:**
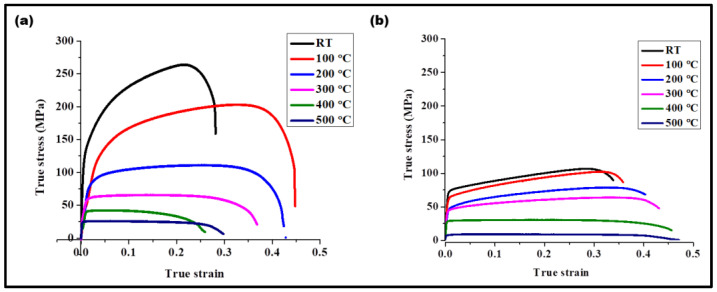
High-temperature tensile tests of the (**a**) composite and (**b**) processed without PDC from room temperature to 500 °C. The tensile strength of the composite at 100 °C was two times that of the room temperature strength of metal processed without PDC, along with an increase in ductility of 25%.

To retain strength at high temperatures, the microstructural features need to have sufficient barriers for dislocation movement. Grain boundaries, particles within the matrix, dislocation substructures are barriers for dislocation motion. Tensile tests for the composite were carried out from 100 °C to 500 °C and the results are presented in Figure 10. At the temperature of 200 °C, the tensile strength of the composite was comparable with the tensile strength of the processed base metal without the PDC at room temperature. Further, the tensile strength of the composite at 100 °C was twice that of the strength of the processed base metal without the PDC and with an increase in ductility of 25%. However, above 200 °C, a drop in strength and ductility was observed. The drop in strength was due to an increased rate of recovery via the process of dislocation annihilation by climb. It is reported that the dislocation loops travelling in parallel slip planes can be annihilated by either addition of atoms or vacancies, depending on the sign of interacting dislocations. Further, interaction and intersection of the dislocations leads to dislocation jogs. The jogged dislocation can move by producing point defects, i.e., vacancies or interstitials [36]. Non-conservative motion of jogged dislocations takes place via vacancy diffusion. Therefore, additional vacancies can be generated during the high temperature deformation.

**Figure 11:**
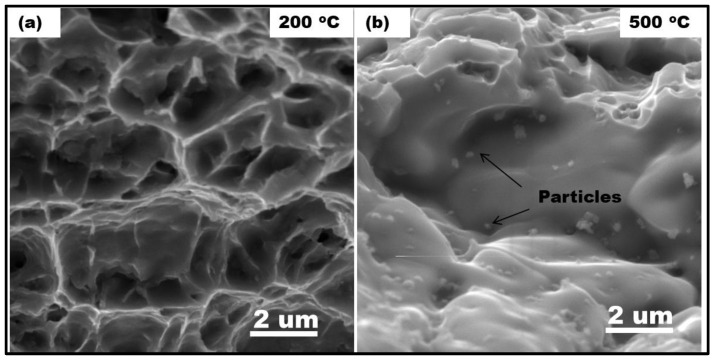
Fracture surface of the sample tensile tested (**a**) at 200 °C and (**b**) at 500 °C. Particles are visible on the fracture surface at 500 °C, indicating that failure took place at the particle–matrix interface.

The tensile failure at high temperatures in this study may be due to a combined effect of void nucleation and growth owing to the generation of additional vacancies via the motion of jogged dislocations and the interface between the particle and matrix becoming weak via thermal expansion mismatch. Figure 11 shows the fracture features of the composite (a) at 200 °C and (b) at 500 °C. At 200 °C, the fracture was purely ductile, with large dimples. Particles were not visible on the fracture surface. Therefore, the fracture took place through the matrix. The bond between the particles and the matrix was stronger than the matrix itself. Conversely, many particles were visible on the fracture surface of the sample tested at 500 °C. This indicates that failure took place at the interface between the particle and matrix at this temperature. As the particles were located not only at grain boundaries but also in grain interiors, the failure was not necessarily a grain boundary failure process. Nevertheless, at 500 °C, grain boundary shearing could also contribute to the damage of the grain boundaries, promoting failure at the boundaries. In that case, however, the particles were supposed to fall out from the damaged boundaries, while they remained in place according to Figure 11b. It is therefore more probable that failure was promoted by the weakening of the bonding between all the kinds of particles and the matrix, irrespective of their location. The occurrence of failure at the particle and matrix interphase with higher temperatures led to an earlier fracture, i.e., lower ductility, due to weakening of the interfacial bonding between the particle and matrix with the increasing temperature.

#### 3.2.2. Thermal Stability up to 500 °C

**Figure 12:**
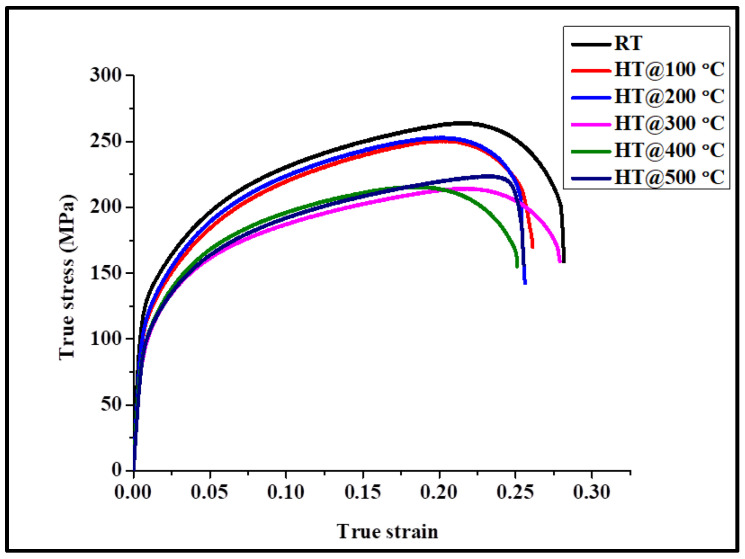
Tensile tests at room temperature of the composite after exposure to temperatures ranging from room temperature to 500 °C over 1 h. The drop in strength was only 15% after exposure at 500 °C.

Figure 12 shows the tensile curves obtained at room temperature for samples exposed to temperatures in the range from room temperature to 500 °C for 1 h. It is apparent that there is not much of a loss in strength or ductility even after high-temperature exposure up to 500 °C. The reduction in ultimate tensile strength (UTS) was only 4% after exposure to 100 °C, 17% to 300 °C, and 15% to 500 °C for 1 h. The loss in strength is much higher in commercial high-strength aluminum alloys; for example, in Al7075-T6 alloy, the loss is 50% at 300 °C [37] in contrast to 17% in our material. This is why–in spite of the higher UTS in Al7075-T6–the UTS becomes comparable between the two materials after a 300 °C 1 h exposure: 225 MPa in our material compared to 258 MPa in Al7075-T6. The higher rate of loss in traditional high-strength Al-based alloys with high-temperature exposure is due to precipitate coarsening/dissolution and grain growth.

**Figure 13:**
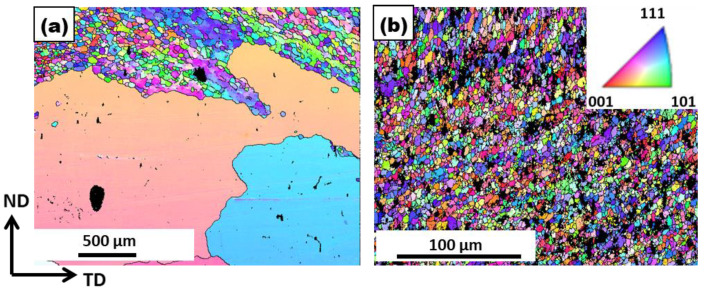
SEM-EBSD micrograph of the (**a**) processed metal without PDC and (**b**) composite after heat treatment at 500 °C for 1 h. Abnormal grain growth is observed in the processed metal without PDC.

**Figure 14:**
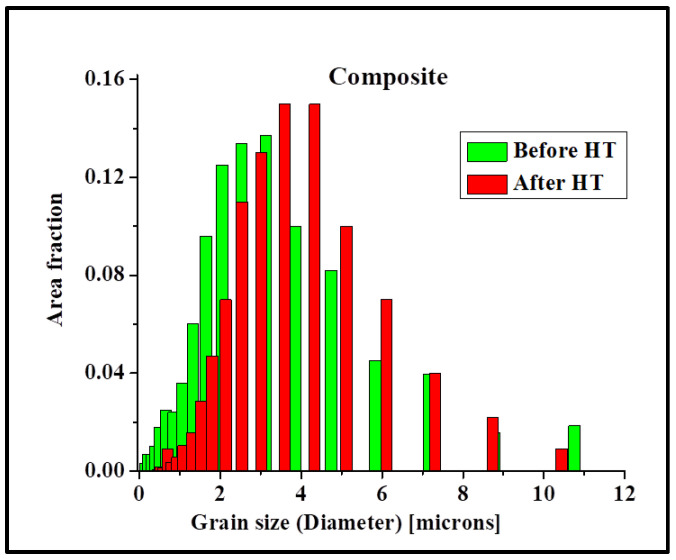
Grain size distribution of the composite before and after heat treatment. The average grain size remained the same; however, individual grains changed their size.

To study the grain growth in the composite, microstructural examinations were carried out at the highest temperature of 500 °C. During FSP, the grain boundary area increases due to grain refinement, thus increasing the energy stored in the material. When such materials are subjected to high temperatures, the stored energy is reduced by grain growth. Figure 13a,b shows the grain structure of the processed metal with and without the PDC after heat treatment at 500 °C for 1 h. Abnormal grain growth was observed in the sample processed without the PDC. No change in average grain size (4 μm) of the composite before and after heat treatment was observed. The microstructure seemed very much the same after the heat treatment. It is not excluded from consideration, however, that individual grains changed their size. This aspect is confirmed by comparing the grain size distribution before and after heat treatment as shown in Figure 14. The sample without the PDC particles exhibited significant grain growth, while the Al-PDC composite showed very high thermal stability. The mechanical properties were also retained along with grain stability. In contrast, precipitation-hardened alloys lose their properties above 200 °C due to precipitate dissolution and coarsening at higher temperatures.

**Figure 15:**
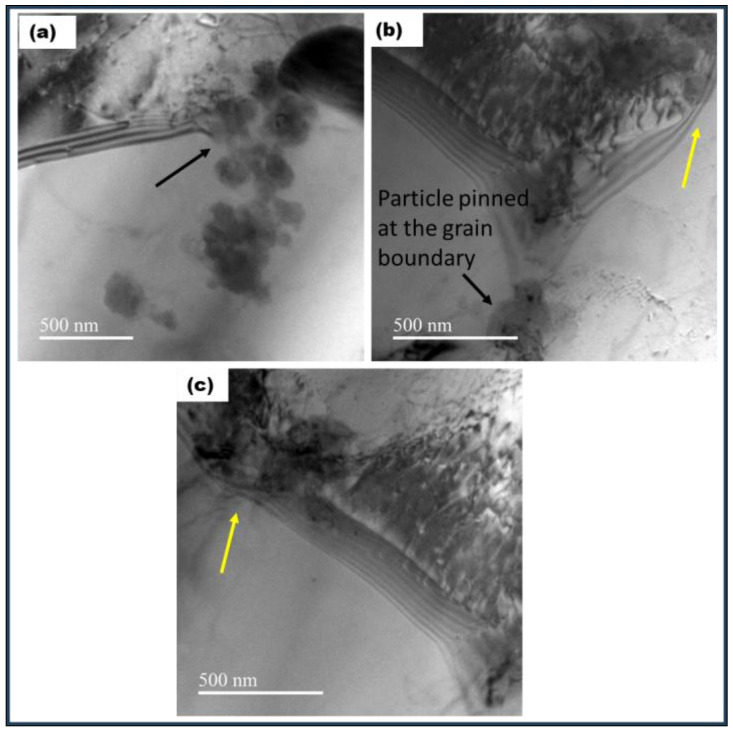
(**a**): Bright-field TEM micrograph showing particles at the grain boundary (marked with a black arrow). (**b**,**c**): grain boundary drag at the particle–boundary interface is seen (marked with a yellow arrow).

The bright-field TEM micrographs presented in Figure 15 show particles pinning at the grain boundaries. These particles provide a large drag force to the grain boundaries and impede their motion at high temperatures.

In summary, a composite was produced by incorporating polymer-derived ceramics as reinforcement into an aluminum matrix. The composite exhibited superior mechanical properties and thermal stability. Furthermore, significant strain hardening was measured, which increases ductility. The occurrence of both strain hardening and the Bauschinger effect shows that dislocation looping around the reinforcements was the dominant mechanism. We believe that by controlling the volume fraction, size, and distribution of these reinforcements, one can obtain the required properties in a single class of material, thus making recycling easier.

## 4. Conclusions

In this present work, Al-PDC composite was successfully produced via FSP. The samples were characterized for their microstructure and tensile properties. The following salient features can be drawn from the results:

1. A new Al-PDC composite was prepared via friction stir-processing. The composite exhibited a 2.5-fold increase in yield strength and 3.5-fold increase in ultimate strength.
2. The ceramic particles incorporated were amorphous and incoherent. The increase in strength was due to the particles acting as strong barriers to dislocation motion. The dislocation bowing mechanism dominated, which manifested in strain hardening and an increase in the Bauschinger effect.
3. The composite retained mechanical properties up to 200 °C. Above that, the properties started to decline. The presence of particles on the fracture surface of the sample tested at 500 °C indicated that the particle–matrix bonding become weak, and the failure took place at the particle–matrix interface.
4. The composite exhibited grain stability when exposed to 500 °C for one hour. This effect is due to particle pinning of the grain boundaries via the Zener mechanism.

## Data Availability

More experimental data can be requested from the authors.
